# Retraction Note: Small interfering RNA targeted to stem-loop II of the 5′ untranslated region effectively inhibits expression of six HCV genotypes

**DOI:** 10.1186/s12985-021-01623-y

**Published:** 2021-07-26

**Authors:** Ramesh Prabhu, Robert F. Garry, Srikanta Dash

**Affiliations:** 1 Department of Pathology and Laboratory Medicine, Tulane University Health Sciences Center, 1430 Tulane Avenue, New Orleans, LA70112 USA; 2 Department of Microbiology and Immunology, Tulane University Health Sciences Center, 1430 Tulane Avenue, New Orleans, LA 70112 USA

## Retraction Note: Virology Journal 2006, 3:100 10.1186/1743-422X-3-100

The Editor-in-Chief has retracted this article. Concerns were raised regarding a number of figures, specifically:

- Figure 4: the pCVH77C/+siRNA74 panel appears to overlap with the pCVJ4L6S/+siRNA74 panel
- Figure 4: the pCVH77C/Full-length HCV panel appears to be identical with the pM.O.9.6.-T7/+EBNA1 panel
- Figure 6: the bottom center and bottom right panels appear to be identical

The article also shows significant overlap with a previously published article [1]. The Editor-in-Chief therefore no longer has full confidence in the reliability of some data reported in the article.

Robert F. Garry and Srikanta Dash agree to this retraction. The editor was not able to obtain a current email address for Ramesh Prabhu.
